# The quantitative motion analysis using portable gait rhythmogram after CSF drainage in the patients with idiopathic normal pressure hydrocephalus

**DOI:** 10.1186/2045-8118-12-S1-P57

**Published:** 2015-09-18

**Authors:** Makiko Yogo, Shusaku Omoto, Masayo Morita, Masahiko Suzuki

**Affiliations:** 1 Department of Neurology, The Jikei University School of Medicine, Katsushika Medical Center, Tokyo, Japan

## Introduction

We previously reported that portable gait rhythmogram(PGR) equipped with accelerometers to identify gait-induced accelerations enable us to quantitative gait analysis in Parkinson's disease (PD). We objected to reveal the quantitative gait differences between idiopathic normal pressure hydrocephalus (iNPH), PD and normal controls (NC) using PGR.

## Methods

The study subjects were 7 patients with iNPH (age 76.9±2.6 years, 6 men and 1 woman), 12 patients with de novo PD who were not medicated with any kinds of anti-parkinsonian drug (age 68.3±4.5 years, 5 men and 7 women, 2 patients with Hoehn and Yahr stage I, 2 with stage II, 8 with stage III. The duration of illness was 2.3±1.6 years.) We also studied 17 NC (age 64.7±4.5 years, 8 men and 9 women).

24hours continuous recordings were performed twice before and after CSF drainage in iNPH patients with a trunk-mounted PGR. In PD and NC, 24hours continuous recordings were performed once at arbitrary point.

We calculated the “amount of overall movements per 24 hrs” from all motion-induced acceleration as an index of hypokinesia, “gait cycle and cadence” from the gait signals as indexes of step length, and “gait acceleration range” as an index of step power.

## Results

Comparing with NC and PD, the average data obtained from 6 CSF drainage responded iNPH patients showed lower “amount of overall movements per 24 hrs”. Faster “gait cycle” and higher “cadence” which indicate narrower step lengths, and lower “gait acceleration range” which indicate ineffective floor reaction forces in iNPH patients were also detected. Improvements in gait following spinal CSF drainage were observed using PGR.

In representative case of CSF drainage responded patients, gait defects improved after CSF drainage, and the improvement lasted after LP shunt was performed. Same tendency were observed in the average result of responded patients, though it was not statistical significant.

Whereas, no alteration was observed after CSF drainage in an un-responded subject.

When considering percent changes of parameters, positive correlations were found between “amount of overall movements” and points of FAB, between “cadence” and points of MMSE, between “cadence” and 10m walk time. Negative correlation was found in “amount of overall movements” and 10m walk time, between “gait acceleration range” and points of FAB.

## Conclusions

PGR revealed narrower and more monotonous step length in iNPH patients than PD and NC. And it also detected gait improvements following CSF drainage in the CSF drainage responded patients. Thus, PGR can analyze long durational gait in daily life and provide additional quantitative data on former measurements of CSF drainage easily and usefully.

